# Super-resolution photoacoustic and ultrasound imaging with sparse arrays

**DOI:** 10.1038/s41598-020-61083-2

**Published:** 2020-03-13

**Authors:** Sergey Vilov, Bastien Arnal, Eliel Hojman, Yonina C. Eldar, Ori Katz, Emmanuel Bossy

**Affiliations:** 1 Univ. Grenoble Alpes, CNRS, LIPhy, 38000 Grenoble, France; 2 Department of Applied Physics, Hebrew University of Jerusalem, 9190401 Jerusalem, Israel; 3 Faculty of Mathematics and Computer Science, Weizmann Institute of Science, Rehovot, Israel

**Keywords:** Photoacoustics, Imaging and sensing

## Abstract

It has previously been demonstrated that model-based reconstruction methods relying on *a priori* knowledge of the imaging point spread function (PSF) coupled to sparsity priors on the object to image can provide super-resolution in photoacoustic (PA) or in ultrasound (US) imaging. Here, we experimentally show that such reconstruction also leads to super-resolution in both PA and US imaging with arrays having much less elements than used conventionally (sparse arrays). As a proof of concept, we obtained super-resolution PA and US cross-sectional images of microfluidic channels with only 8 elements of a 128-elements linear array using a reconstruction approach based on a linear propagation forward model and assuming sparsity of the imaged structure. Although the microchannels appear indistinguishable in the conventional delay-and-sum images obtained with all the 128 transducer elements, the applied sparsity-constrained model-based reconstruction provides super-resolution with down to only 8 elements. We also report simulation results showing that the minimal number of transducer elements required to obtain a correct reconstruction is fundamentally limited by the signal-to-noise ratio. The proposed method can be straigthforwardly applied to any transducer geometry, including 2D sparse arrays for 3D super-resolution PA and US imaging.

## Introduction

Ultrasound^1^ (US) and photoacoustic^2^ (PA) imaging are now widely applied biomedical imaging modalities. They both usually use multielement transducer arrays as ultrasonic detectors for acquiring acoustic signals. Developed for two-dimensional (2D) or cross-sectional imaging, linear transducer arrays are widely spread in research and clinical applications. For three-dimensional (3D) single shot imaging, 2D array matrices should be used instead of linear arrays. However, the availability of 3D imaging equipment is first limited by the sophisticated fabrication process involved: probes for 3D imaging may have several thousands of elements to connect which makes the assembly of such probes technically difficult^3^. Second, to control simultaneously as many elements as possible, sophisticated ultrasound electronics with a very large number of channels are needed. Decades ago, sparse arrays were proposed^4,5^ to reduce substantially the number of transducer elements required for 3D US imaging. The corresponding reduction scheme suggests using a selection of elements of a dense periodic array without changing the total transducer aperture. Such a selection can either be a random subset of elements^4^ or a defined pattern^5^. Experimental investigations of imaging performances of sparse arrays in US imaging showed that sparse arrays can provide a diffraction-limited resolution similar^6,7^ to that of all-element arrays. Special reconstruction techniques have been recently proposed for sparse array US imaging. In particular, the convolutional beamforming algorithm was reported^8^ to provide better (although still diffraction-limited) resolution than standard delay-and-sum beamforming. It is worth noting that in radar technologies it has also been shown that advanced compressed sensing methods^9^ permit preserving the diffraction -limited resolution when using radars with a reduced number of elements^10,11^.

Sparse arrays have also been widely applied in 3D photoacoustic tomography^12–18^. In most of these studies sparse arrays were used to obtain diffraction-limited images with model-based reconstruction^13–18^. The model-based reconstruction approach considers a forward linear model expressed as *S* = ***A****T*, where *S* are the acquired signals, *T* is the object to reconstruct, and the propagation matrix ***A*** is a library containing the point spread function (PSF) at all points of the discretized imaging zone. The reconstruction consists in finding the object that minimizes a cost function defined as the sum of a fidelity term (taking into account the measurement data and the model) and a regularization term (taking into account measurement noise and prior knowledge on the object).

As the main novelty of this work, we investigate the possibility of using sparse ultrasound arrays in the context of *super-resolution* imaging with the model-based approach. The classical resolution in both PA and US imaging of biological tissues at depth is limited by acoustic diffraction. Specifically, it is the absorption of ultrasound in tissue that limits the highest ultrasound frequency detectable at a given depth, and therefore the resolution via the diffraction phenomenon. In particular, the depth-to-resolution ratio turns out to be of the order of 100 for both US and PA imaging of biological tissue. As an example, for a 10 MHz US probe, that can be ordinarily used for US or PA imaging at a few centimeters depth in tissues, diffraction limits the resolution to 100-200 *μ*m in typical imaging conditions^19^.

Imaging beyond the diffraction limit has first been investigated in optical microscopy, resulting in the advent of Nobel-prize winning pioneering methods such as photo-activated localization microscopy (PALM)^20^ and stimulated emission depletion (STED) microscopy^21^. Following the aforementioned advances in optics, several super-resolution techniques have been proposed more recently for both US and PA imaging. Super-resolution methods include localization-based imaging, fluctuation-based reconstruction and model-based reconstruction. As for optical imaging, the localization approach in US^22–25^ and PA^26,27^ imaging relies on the detection of individual scatterers or absorbers. The idea is that the coordinates of a point-like source can be determined with a precision much better than the size of the imaging PSF provided that this PSF can be separated from those of the other sources in some parameters space. This separation condition imposes a low concentration of sources, and therefore the use of contrast agents for visualization of biological structures. Fluctuation-based PA^28,29^ and US^30^ techniques, based on the principles of super-resolution optical fluctuation imaging (SOFI), exploit uncorrelated fluctuations from different sources. While fluctuation-based approaches eliminate the need for isolating individual sources, they remain limited in terms of spatial resolution improvement and temporal resolution. It was also shown that model-based reconstruction with sparsity constraints on the sample, an approach originating from the field of compressed sensing^9^, could yield super-resolved US^31,32^ and PA^33^ images. A major advantage of model-based reconstruction over the previously mentioned localization-based and fluctuation-based techniques is that by requiring (in principle) only a single-shot acquisition it permits a high temporal resolution. Some very recent works^34–36^ have proposed to mix fluctuation-based and model-based techniques to achieve super-resolution in US^36^ and PA imaging^35^. In PA imaging, it was also demonstrated^34^ that super-resolution can be obtained via the joint support recovery through the model-based approach. In particular, the vector *S* is composed of data measured for several PA acquisitions, with each acquisition corresponding to a random speckle illumination of *the same* absorbing structure forming the joint support.

In US imaging, the use of 2D sparse arrays for super-resolution imaging has recently been demonstrated by use of a localisation approach and microbbubles^25^. Here, we investigate the possibility to perform *both* US and PA super-resolution imaging of *sparse samples* with a label-free model-based reconstruction approach using a *sparse array*. More specifically, we apply sparsity-constrained model-based reconstruction to perform 2D super-resolution imaging of sparse test samples with only 8 (out of 128) elements of a linear transducer array. We show that this reconstruction approach can be used for both PA and plane-wave US imaging with the same experimental setup. To build the matrix ***A*** that describes the forward model, we propose a novel method that involves only one PSF acquisition, as opposed to the measurement of the full set of PSFs in the imaging zone. We also report simulation results showing how the reconstruction quality is related to the number of transducer elements and the signal-to-noise ratio (SNR).

## Results

**Figure 1:**
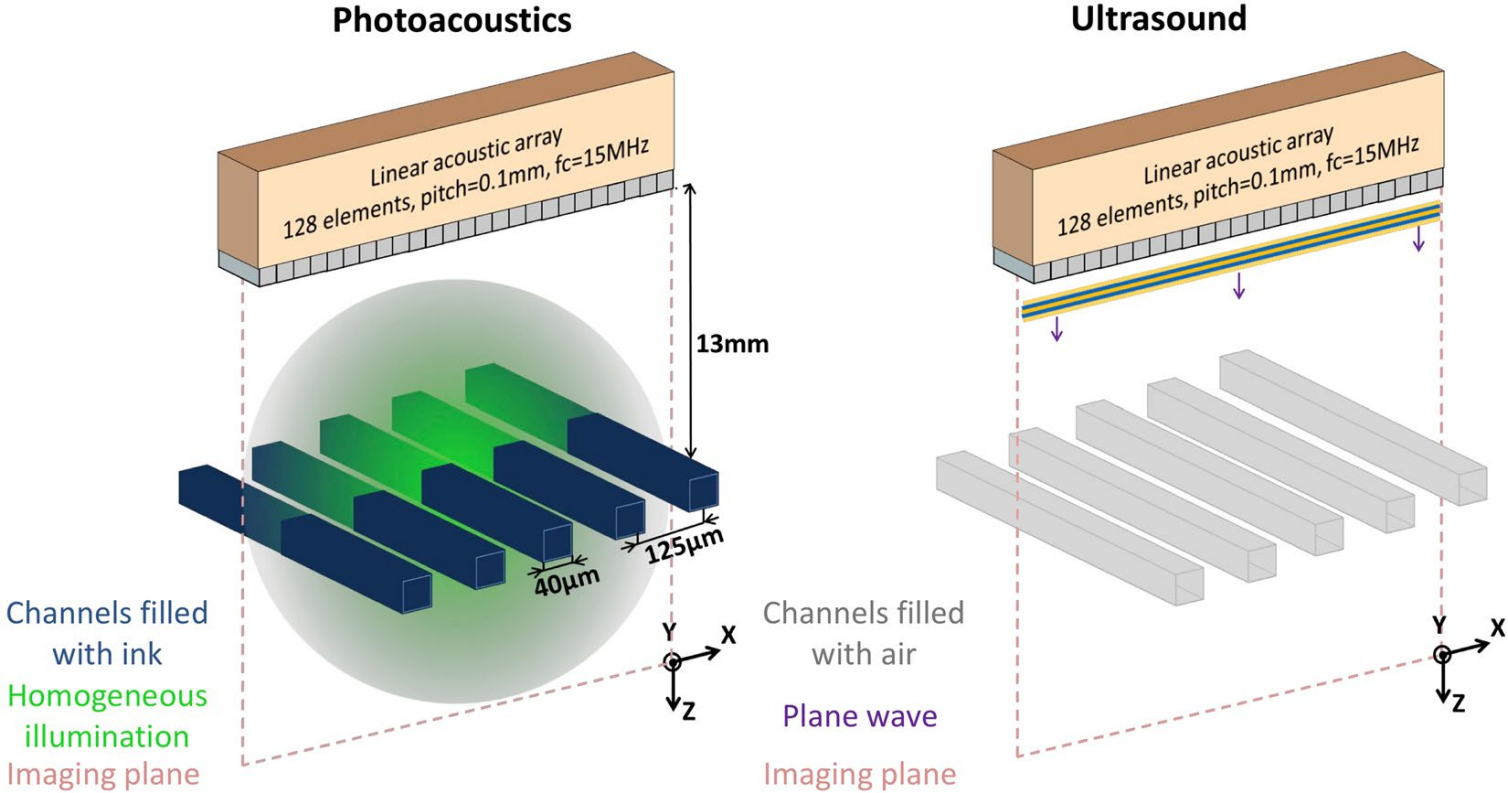
Experimental setup. Samples carrying 5 parallel microchannels are placed perpendicularly to the imaging plane. Each channel is 40 *μ*m wide and 50 *μ*m deep, the center-to-center interchannel distance being *L*_*cc*_ = 125 *μ*m. In the PA experiment, the sample is illuminated by a laser pulse and the resulting PA signals are detected by a linear US probe. In the US experiment, a plane wave is emitted in the sample direction and the backscattered signals are collected. The purple arrows indicate the propagation direction of the ultrasound plane wave.

To demonstrate that sparsity-constrained model-based reconstruction can provide super-resolution in sparse-array PA or US imaging, we carried out two proof-of-concept experiments (one for US and one for PA imaging). The goal of each experiment was to recover a super-resolved cross-sectional image of a sparse five-channel microfluidic sample by processing data received by different subsets of elements of a linear ultrasound probe. The imaging configuration for both experiments is shown in Fig. 1 and a picture of the setup is available in Supplementary Materials. Importantly, in both PA and US experiments, we used the same imaging equipment, the same acquisition geometry, samples of identical structure, and the same reconstruction method. Only the nature of the contrast and the way the ultrasound wave is generated were different in these experiments. In the PA experiment, the microfluidic channels were filled with absorbing liquid and illuminated by pulsed light. In the US experiment, the microfluidic channels were filled with air and a plane ultrasound wave was emitted by the transducer array. In both experiments, the measurement data consisted of the ultrasound signals detected by the array for a single shot excitation (light pulse or plane wave). Prior to imaging the five-channel samples, the PSF required to build the forward model was measured for each type of sample (absorber-filled or air-filled) in the corresponding imaging mode (PA or US).

The image reconstruction consisted in finding the object that minimizes a cost function. This cost function was based on the forward model (derived from the PSF calibration) and a l_1_-norm regularization term used to suppress the measurement noise and select a sparse object. This model-based reconstruction was then compared to the conventional delay-and-sum approach taken as a typical diffraction-limited reconstruction approach. Further details on the experimental and reconstruction methods can be found in the Methods section.

**Figure 2:**
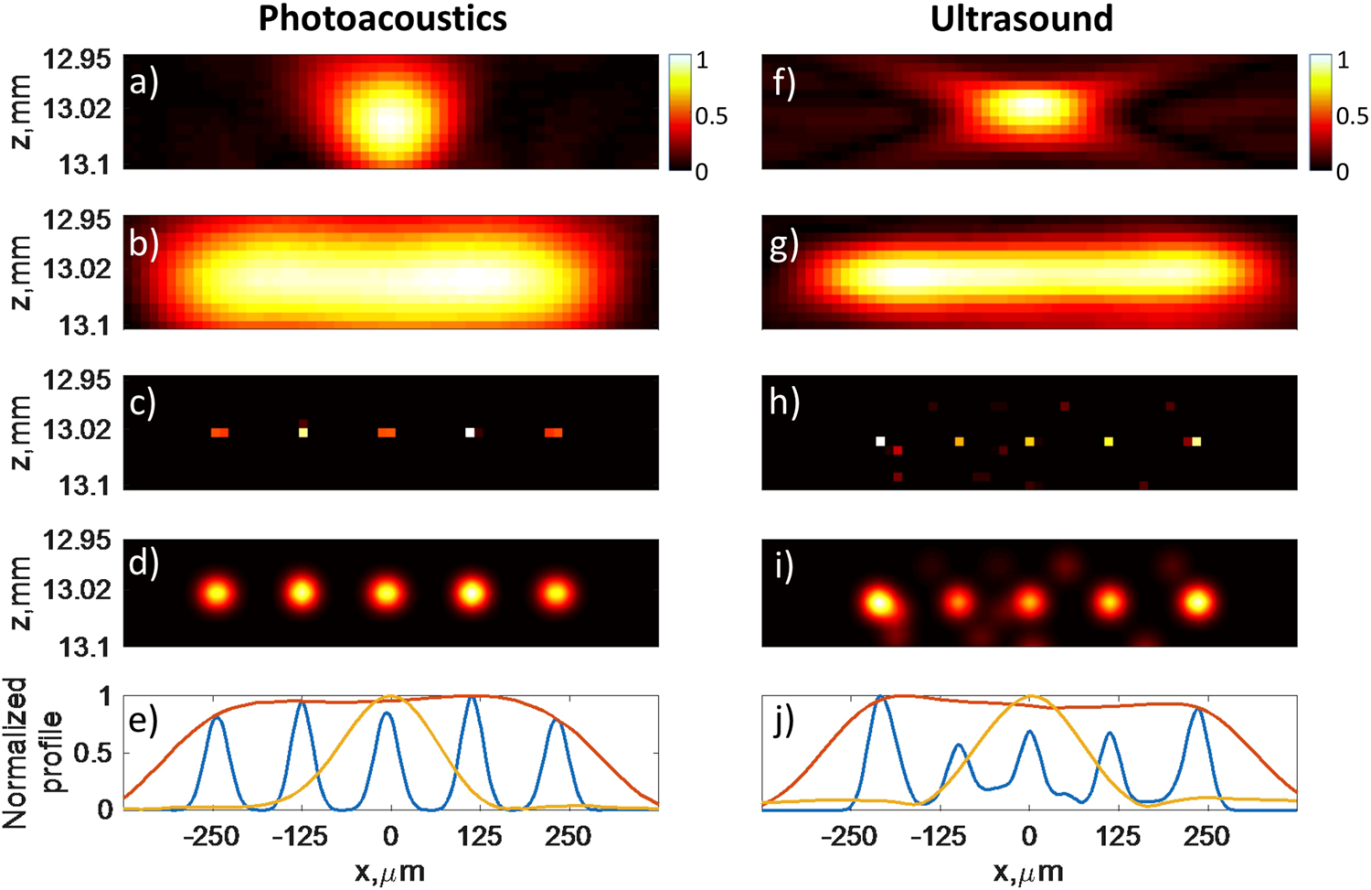
Experimental images reconstructed in the PA (**b–e**) and US (**g–j**) experiments alongside with the corresponding PSFs (**a,f**). All the 128 elements of the probe were used. (**b,g**) Conventional beamforming cross-sectional images of the sample. The microchannels are indistinguishable as the center-to-center distance *L*_*cc*_ = 125 *μ*m is smaller than the lateral FWHM of the PSF (155 *μ*m). (**c,h**) Images obtained with model-based reconstruction. The reconstruction recovers five distinct regions corresponding to the microfluidic channels. (**d,i**) Reconstruction images (**c,h**) after smoothing out by a 2D spatial Gaussian filter (*σ* = 12.5 *μ*m) and interpolating on a 3.125 *μ*m grid. (**e,j**) Yellow line: normalized amplitude profile on the PSF envelope images (**a,f**); red line: normalized amplitude profile on the envelope images (**b,g**); blue line: normalized amplitude profile on the filtered reconstruction images (**d,i**).

We first demonstrated for both PA and US imaging that sparsity-based reconstruction led to super-resolution images of sparse samples when using all the 128 available elements of the probe. The conventional delay-and-sum images are shown in Fig. 2b,g. It can be noticed that the conventional reconstruction is affected by diffraction-limited resolution: two neighboring channels can not be separated, resulting in a bar-like pattern in place of the five individual channels. The center-to-center distance between neighbouring channels (*L*_*cc*_ = 125 *μ*m) is indeed below the diffraction limit defined by the lateral full width of half maximum (FWHM) of the PSF (measured to be 155 *μ*m). Meanwhile, the five individual channels are clearly resolved in both PA and US sparsity-constrained model-based reconstruction images (Fig. 2c–e,h–j).

**Figure 3:**
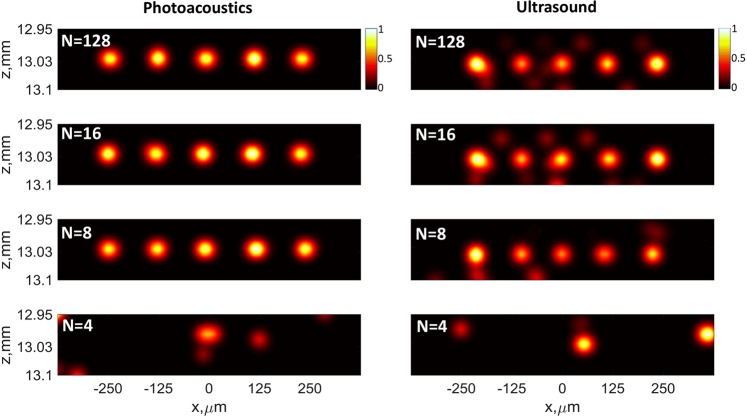
Images obtained with model-based reconstruction in PA and US experiments using *N* = 4, 8, 16 and 128 transducer elements regularly distributed along the probe aperture. A 2D spatial Gaussian filter (*σ* = 12.5 *μ*m) and interpolation on a 3.125 *μ*m grid were applied after the reconstruction.

Then, as a major result of this work, we demonstrated that it is also possible to obtain similar super-resolved images using a sparse array in the place of the initial 128-element dense array. To emulate sparse-array imaging, we applied the same reconstruction approach to data acquired by only a fraction of the probe’s elements. To maintain the same conventional diffraction limit, the resulting probe aperture was kept constant by including the first and the last elements of the probe. The other elements were regularly distributed along the probe. The objects reconstructed with 16, 8 and 4 elements are compared to the full-probe (128 elements) reconstruction in Fig. 3.

The images in Fig. 3 show that in both PA and US experiments the five channels can be imaged using down to only 8 array elements. With less than *N*_*min*_ = 8 elements, we were not able to reconstruct the imaged structure properly. Random distributions of the transducer elements were also considered, but no significant differences in the results were found as compared to the linear distribution case (See Supplementary Figs. 2 and 3).

**Figure 4:**
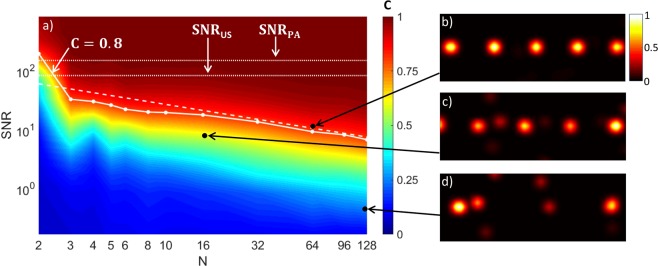
Simulation results. (**a**) - Correlation *C* as a function of the SNR and the number of transducer elements *N*, dotted lines: *SNR*_*PA*_ = 150 in the PA experiment, *SNR*_*US*_ = 83 in the US experiment, dashed line: fitting function SNR$\propto \sqrt{N}$ for *C* = 0.8, (**b–d**) - typical simulation images: (**b**) - *N* = 64, SNR = 16, *C* = 0.85, (**c**) - *N* = 16, SNR = 10, *C* = 0.57, (**d**) - *N* = 128, SNR = 0.8, *C* = 0.23.

In order to shed some light on the parameters that condition the minimal number *N*_*min*_ of transducer elements needed to yield a faithfull reconstruction, we carried out a series of numerical simulations. In these simulations, as a measure of the reconstruction quality, we studied the correlation *C* between the reconstructed object and a modelled ideal object, as a function of the number of transducer elements and the signal-to-noise ratio (SNR). In this work, we define the SNR as the ratio between the peak amplitude of the radio frequency (RF) signal and the standard deviation of the measurement noise computed over a signal-free region of the RF data. The simulation results are illustrated in Fig. 4. As could be intuitively expected, these results show that for any fixed number *N* of transducer elements involved in the reconstruction, the reconstruction image quality increases with the SNR. Moreover, the SNR that assures a given reconstruction quality approximately scales as the square root of the number of elements, apart from *N* = 2. This is illustrated for *C* = 0.8 in Fig. 4 with the dashed line following SNR$\propto \sqrt{N}$. For *N* = 2, our simulations still predict the possibility to reconstruct the imaged object, but with a much stronger requirement on the SNR.

## Discussion

Previous studies reported super-resolution images obtained via model-based reconstruction with sparsity priors on the imaged object, in the field of US imaging^31,32^ and more recently in PA imaging^33^. In this work, we first illustrated the generality of the sparsity-constrained model-based approach by applying this method to obtain both experimental PA and US images of sparse test objects, with the same imaging equipment being used in the PA and US experiments. As a key result of the present work, we demonstrated that imaging sparse objects beyond the acoustic diffraction limit remains possible even when a very small number of transducer elements are used for the reconstruction, much below the number required by Nyquist spatial sampling. This suggests that in imaging with conventional number of transducer elements, a significant part of the measurement information might be redundant. By means of numerical simulations, we showed that if the SNR is very high then by reconstructing images with a perfectly known forward model it is possible to obtain the correct object reconstruction with only two transducer elements. According to the simulation results, more than two elements are necessary to provide the correct reconstruction in the case of typical experimental values of the SNR: for a given SNR on each transducer element, using more elements may be considered equivalent to reducing the influence of the measurement noise. In practice, additional uncertainties of the forward model might further limit the reconstruction quality: in our experiments the minimal number of elements *N* = 8 required for the reconstruction with correlation *C* = 0.8 was above the number of elements *N* = 3 predicted by the simulations for the same SNR and the same correlation.

In addition, a certain difference in image reconstruction quality between PA images (left columns in Figs. 2 and 3) and US images (right columns in Figs. 2 and 3) can be noticed. In particular, the SNR in the US reconstruction images is lower than in the PA images. In addition, the average interchannel distance in the US images (112 *μm*) slightly deviates from the true value (125 *μm*), whereas in the PA images the interchannel distance is restored correctly. This difference between the PA and US reconstruction images may result from several reasons. First, the SNR in the US experiment was lower than in the PA experiment. Second, the uncertainties of the forward model might have played a greater role in the US experiment.

As a novelty of our work, we derived the forward model by measuring only one PSF, rather than all PSFs in the field of view, which was done, for instance, in^33^. This approach assumes that PSFs differ from each other only by time delays that are calculated based on wave propagation in water. However, in our experiments this condition was not fully satisfied at least due to the presence of a PDMS layer between the sources and the receivers. As the US experiment involves a double crossing of this PDMS layer, it is likely that in the US experiment the forward model is less accurate than in the PA experiment, leading to a less faithful reconstruction image.

In conclusion, we demonstrated experimentally the possibility of performing both PA and US super-resolution imaging of sparse samples with a sparse array (with down to 8 transducer elements) by applying a model-based reconstruction approach. Although the results demonstrated here were obtained in 2D imaging with a 1D linear array, a major advantage of the proposed approach is that it can be applied to any transducer geometry, and therefore in 3D imaging. Provided that the relative positions of the transducer elements are known, the proposed method of constructing the forward model from a single PSF measurement remains valid. It was shown by reconstructing simulated data that the SNR yields a fundamental limit on the number of transducer elements needed to provide a faithful reconstruction. The relative influence of the SNR and the uncertainties of the forward model on the reconstruction quality as well as the ultimate resolution limit are to be further investigated in order to fully understand and probably predict the number of transducer elements required for super-resolution imaging. While we demonstrated that it is possible to obtain super-resolution images of sparse test samples with sparse array, the presented method should be further investigated with more realistic experimental data before firm conclusions can be drawn on its performance in the context of biomedical PA and US imaging.

### Samples

The microfluidic samples were prepared with a standard soft-lithography manufacturing technology^37^ using polydimethylsiloxane (PDMS). Each sample consisted of five hollow channels sandwiched between two layers of PDMS. The thickness of the upper layer (the layer placed closer to the US probe in the experiments) was around 180 *μm*. This thickness was chosen as small as possible since the influence of the PDMS on wave propagation was neglected in the model. Each channel was 40 *μm* wide (x-direction) and 50 *μm* deep (z-direction). The centre-to-centre distance between neighbouring channels was *L*_*cc*_ = 125 *μm*. This distance was deliberately chosen smaller than the estimated lateral full width of half maximum (FWHM) of the PSF (155 *μm*). To acquire the PSF and build the forward model, additional samples containing only one microfluidic channel were prepared, the channel being 10 *μm* wide and 50 *μm* deep. For PA imaging, the channels were filled with an absorbing dye solution (Patent Blue V, absorption peak at 640nm^38^) to provide photoacoustic contrast, while for US imaging the channels were filled with air to provide acoustic contrast.

### Experimental protocol

The imaging configuration used in experiments is illustrated in Fig. 1. The five microfluidic channels were aligned to cross the xz imaging plane perpendicularly, and positioned at the distance *z*_*f*_ = 15 mm from the linear ultrasound probe, the distance *z*_*f*_ corresponding to the elevational focus of the probe. As the ultrasound probe, we used a capacitive micromachined ultrasonic transducer (CMUT) array (L22-8v, Verasonics, USA: *N* = 256 elements with 128 consecutive elements used in our experiments, pitch ≈ 100 *μm*, center frequency *f*_*c*_ ≈ 15 MHz). The probe and sample were immersed in a water tank. In the PA experiment, signals were generated by illuminating the sample with a 5 ns laser pulse (*λ* = 532 nm, fluence ≈ 3 mJ/cm^2^) generated by a frequency-doubled Nd:YAG laser (Spitlight DPSS 250, Innolas Laser GmbH, Krailling, Germany). In the US experiment, a plane ultrasound wave (15 MHz center frequency short pulse) was emitted by the probe, and ultrasound waves backscattered from the sample were recorded. To control emission and reception of ultrasound waves, the probe was connected to multichannel acquisition electronics (High Frequency Vantage 256, Verasonics, USA).

The Vantage system had two 128-channel connectors for US transducers, each connector providing interface for synchronous control of 128 transducer elements. As the transducer used in this work had 256 elements wired to a single probe connector, only 128 of its elements could be accessed simultaneously with our system. Although the whole transducer aperture could in principle be accessed using the internal Vantage multiplexer, this would require two distinct acquisitions. To demonstrate the possibility of single-shot super-resolution as a strong point of the proposed reconstruction approach, we did not use the Vantage system’s multiplexer and restricted our acquisitions to 128 transducer elements.

To reconstruct objects in both PA and US experiments, the PSF was measured by applying the acquisition protocol described above for samples containing a single microfluidic channel. The single channel was placed in the center of the field of view of the ultrasound probe. The conventional lateral resolution defined as the lateral full width at half maximum (FWHM) of the PSF was measured after standard delay-and-sum reconstruction to approximately 155 *μ*m in both PA and US experiments.

### Forward model

Under the assumption of a linear dependence between the quantity to image and the measured radio frequency (RF) signals, the voltage signals measured by the acquisition electronics can be expressed for both PA and US imaging as

1 $$s({t}_{i},{{\bf{r}}}_{k})={\mathbb{A}}[{T}_{0}({\bf{r}})],$$

where *s*(*t*_*i*_, **r**_*k*_) is the signal at time *t*_*k*_ measured by the transducer element located at **r**_*k*_, *T*_0_(**r**) is the quantity to image, and ${\mathbb{A}}$ is a linear operator that takes into account both the ultrasound generation/propagation, and the transducer response. In PA imaging, *T*_0_(**r**) is proportional to the absorption coefficient *μ*_*a*_(**r**), under the assumption of homogeneous light illumination and a homogeneous Gruneisen paramater. In US imaging, *T*_0_(**r**) is related to the distribution of the backscattering coefficient, provided that the single scattering regime is valid.

By discretizing the object to reconstruct on a grid, this forward problem may then be written in a matrix form as

2 $$S={\boldsymbol{A}}{T}_{0},$$

where *S*^*m*×1^ is a vector with all the RF data (m = number of time samples × number of transducer elements), ${T}_{0}^{n\times 1}$ is the discretized version of the quantity to reconstruct (n = number of grid points in the reconstruction zone), and ***A***^*m*×*n*^ is a matrix representing the linear operator. Each of the *n* columns of ***A*** represents the RF response for one of the points $\overline{1\,.\,.\,n}$ of the reconstruction zone, i.e. each column of ***A*** is the vector data corresponding to the signals from a single point source in the imaging zone. The matrix ***A*** therefore contains the responses to each point source and can be considered as a matrix of all the system point spread functions (PSF) in the data space.

The matrix ***A*** can be modelled theoretically or measured experimentally. In some previous works, ***A*** was measured for each point of the reconstruction zone^14,15,33,35^. In our work, the matrix ***A*** is obtained experimentally but we perform a measurement for only one point source. As a point source, we use a single isolated microfluidic channel. All the columns of ***A*** are then derived from this measurement data by assuming that the signals from two distinct point sources differ from each other by their arrival time, but remain otherwise identical in shape. In other words, it is assumed that the impulse response of each transducer element is the same for all point sources in the reconstruction zone, apart from a propagation delay. In our experiments, we also neglect the presence of the upper PDMS layer and therefore only consider wave propagation in water, i.e. in a homogeneous isotropic medium with a constant speed of sound *c* = 1500 *m*∕*s*.

Under the assumptions stated above, when a single PSF is acquired for a source placed at {*x*_*j*_, *z*_*j*_}, any column *i* of the matrix ***A*** can be derived from the acquired RF data by shifting the signals for each transducer element *k* with the following time delay:

3 $$\Delta {t}_{i,j,k}={\delta }_{US}\frac{{z}_{j}-{z}_{i}}{c}+\frac{1}{c}[\sqrt{{({x}_{k}-{x}_{i})}^{2}+{({z}_{k}-{z}_{i})}^{2}}-\sqrt{{({x}_{k}-{x}_{j})}^{2}+{({z}_{k}-{z}_{j})}^{2}}],$$

where {*x*_*k*_, *z*_*k*_} are the coordinates of element *k* of the transducer array, {*x*_*i*_, *z*_*i*_} are the coordinates of point *i* of the reconstruction grid. In US imaging, *δ*_*US*_ = 1 to account for the travel time of the emitted plane wave (*δ*_*US*_ = 0 in PA imaging).

### Image reconstruction

To solve the inverse problem, i.e. to find an estimate $\widehat{T}$ of the solution *T*_0_ to the forward model described above, the sparsity-constrained minimization approach similar to those already reported in previous works^31,33,35^ was used. This approach consists in finding $\widehat{T}$ by solving the following minimization problem:

4 $$\hat{T}=\mathop{argmin}\limits_{T}\{{||S-{\boldsymbol{A}}T||}_{2}^{2}+{\alpha }^{2}||T|{|}_{1}\}.$$

In Eq. 4 the *l*_2_ - term ${||S-{\boldsymbol{A}}T||}_{2}^{2}={\sum }_{p=1}^{m}{(S-{\boldsymbol{A}}T)}_{p}^{2}$ corresponds to the least square fitting of the probed quantity *T* to the acquired data *S*. The regularization *l*_1_ - term ${\alpha }^{2}{||T||}_{1}={\alpha }^{2}{\sum }_{q=1}^{n}|{T}_{q}|$ is used to minimize the influence of the noise in *S* while selecting a sparse solution to underdetermined system 2. Regarding typical sizes involved in the reconstruction, for reconstruction with 128 transducer elements the length of the vector *T*^*n*×1^ was *n* = 793 (number of reconstruction points in the field of view), the length of the vector *S*^*m*×1^ was *m* = 4608 (the number of transducer elements multiplied by the number of time samples for each element), the rank of the matrix ***A*** was rank(*A*) = 346, this rank being computed using the standard MATLAB function with the default value of the tolerance parameter. The rank of the matrix ***A*** being inferior to the number of unknowns *n*, system (2) was undetermined. The minimization operation described by Eq. 4 was performed using a fast iterative shrinkage-thresholding algorithm (FISTA)^39–41^. The object to image was reconstructed over a cartesian grid with a *L*_*cc*_/10 = 12.5 *μ*m step. As in previous works^31,33,35^, the value of the regularization parameter *α* was determined heuristically such that the reconstruction image was qualitatively close to the real sample. A 2D Gaussian filter (kernel size *σ* = 12.5*μ*m) and interpolation were applied to improve image visualization. The final image definition corresponds to a grid step of 3.125*μm*. Each reconstruction image was linearly normalized at the final stage via division of each pixel value by the maximum of the intensity in the initial reconstruction image.

The gaussian filter was applied to the reconstructed images only to smooth them, as usually done for other approaches that may lead to point-like reconstructed structures (such as localization based super-resolution). The size of the filter has an influence on the apparent size of individual structures (the microchannels in our case). l_1_-minimization leads to point-like structure, as illustrated in Fig. 2c,h, which show that the size of the channels is not reconstructed correctly. However, the filter does not influence the resolution (defined as the ability to distinguish individual structures), as long as the filter size is smaller than the resolution limit of the method (here close to the distance between neighbouring channels). Here, the filter kernel size *σ* = 12.5 *μ*m is chosen to smooth out the reconstructed object without affecting the ability to distinguish channels, but it should be kept in mind that the apparent size of the channels on the filtered image depends on this choice.

### Simulations

Numerical simulations were performed to produce test data corresponding to imaging five point sources. The stated assumptions on our forward model were strictly followed. Specifically, we modelled the signals received on each transducer element as a time-delayed version of the same signal (simulated for five point sources), based on the delay law described by Eq. 3. The detected signals had a central frequency and bandwidth corresponding to those used in experiments and sampled at the same frequency as in experiments. Simulation data with different SNR values was generated by varying the amplitude of the modelled PA signals, for a fixed noise value. In all simulations, Gaussian noise with a zero mean and a rms of *σ*_*n*_ = 30 was added to the detected signals. Such noise corresponded to the noise produced by the acquisition electronics in our experiments. The simulated distribution of sources was then reconstructed following the same methods as used to reconstruct images from the experimental data. As a metrics of the reconstruction quality, we computed a normalized spatial cross-correlation between each reconstructed object *T* and the ideal reconstruction *T*_*true*_.

The ideal reconstruction *T*_*true*_ was modeled by asserting the value of 1 to the 5 cells of the reconstruction grid corresponding to the positions of the 5 simulated sources. Then, the filtering and interpolation used for the reconstruction images were also applied to *T*_*true*_. In Fig. 4, the correlation *C* for each SNR was estimated by averaging over 100 noise realizations. For each reconstructed image $\widehat{T}$ the correlation *C* was computed using Eq. 5:

5 $$C=\frac{{\sum }_{i=1}^{n}\widehat{T}(i)\cdot {T}_{true}(i)}{\sqrt{{\sum }_{i=1}^{n}{\widehat{T}}^{2}(i)\cdot {\sum }_{i=1}^{n}{T}_{true}^{2}(i)}}.$$

## Supplementary information

Supplementary Information.
