# Supplementary material for: Super-resolution photoacoustic and ultrasound imaging with sparse arrays
Source: Sci Rep. 2020 Mar 13;10:4637. doi: 10.1038/s41598-020-61083-2 (PMC7069938; doi:10.1038/s41598-020-61083-2)
Supplement: Supplementary file 1 — Supplementary Information. [file 41598_2020_61083_MOESM1_ESM.pdf]

Super-resolution photoacoustic and ultrasound imaging with sparse arrays.

Supplementary Information.

Sergey Vilov[1], Bastien Arnal[1], Eliel Hojman[2], Yonina C. Eldar[3], Ori Katz[2], and Emmanuel Bossy[1,*]

[1]Univ. Grenoble Alpes, CNRS, LIPhy, 38000 Grenoble, France
[2]Department of Applied Physics, Hebrew University of Jerusalem, 9190401 Jerusalem, Israel
[3]Faculty of Mathematics and Computer Science, Weizmann Institute of Science, Rehovot, Israel
[*]emmanuel.bossy@univ-grenoble-alpes.fr

Experimental setup

A scheme of the PA and US experiments is shown in the main text (Figure 1). In Supplementary Figure 1, we provide a picture of the experimental setup to give the reader a better idea of how the experiments were conducted.

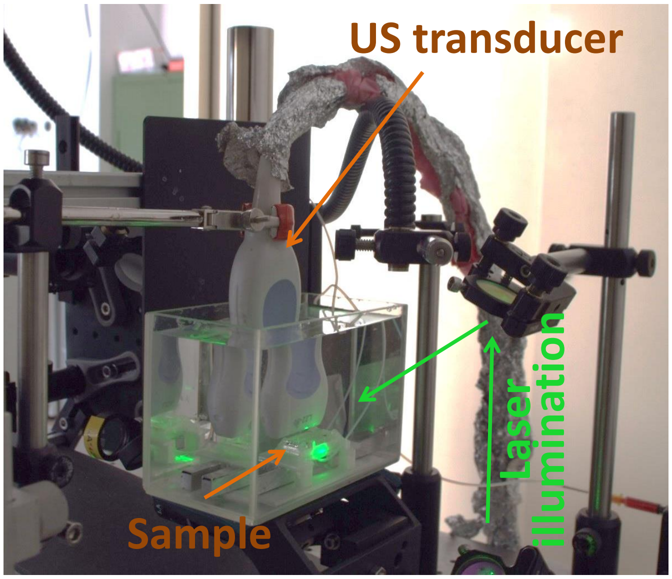

Supplementary Figure 1. Experimental setup used in the US and PA experiments. In the PA experiment, the collimated laser beam (diameter D $\approx$ 10 mm) is reflected by the mirror in the direction of the sample residing on the 3D printed plastic support. This beam illuminates the sample, placed perpendicularly to the imaging plane XZ of the US transducer, at 45°.

Random subsets of elements

To test whether the way of distributing elements along the probe aperture has an impact on the reconstruction quality, we generated several irregular distributions with the total of 32 and 8 elements (Supplementary Figures 2(a) and 3(a) correspondingly). In all random distributions the two border elements were included in order to preserve the classical resolution limit. Then, for each irregular distribution and each value of the SNR the average correlation C was computed following the procedure described in the Simulations section of the article.
The obtained correlation C as a function of the SNR for the generated subsets of 32 and 8 elements is illustrated in Supplementary Figures 2(b) and 3(b) correspondingly. As for the regular distributions, a higher SNR is needed to achieve given correlation with 8 elements than with 32 elements. However, there is little difference between the correlation values for regular and

irregular distributions of transducer elements. The most probable reason is that all the changes in the elements positioning are automatically taken into account by the propagation matrix $\boldsymbol{A}$. As a result, the proposed reconstruction is virtually insensitive to the elements distribution along the probe aperture.

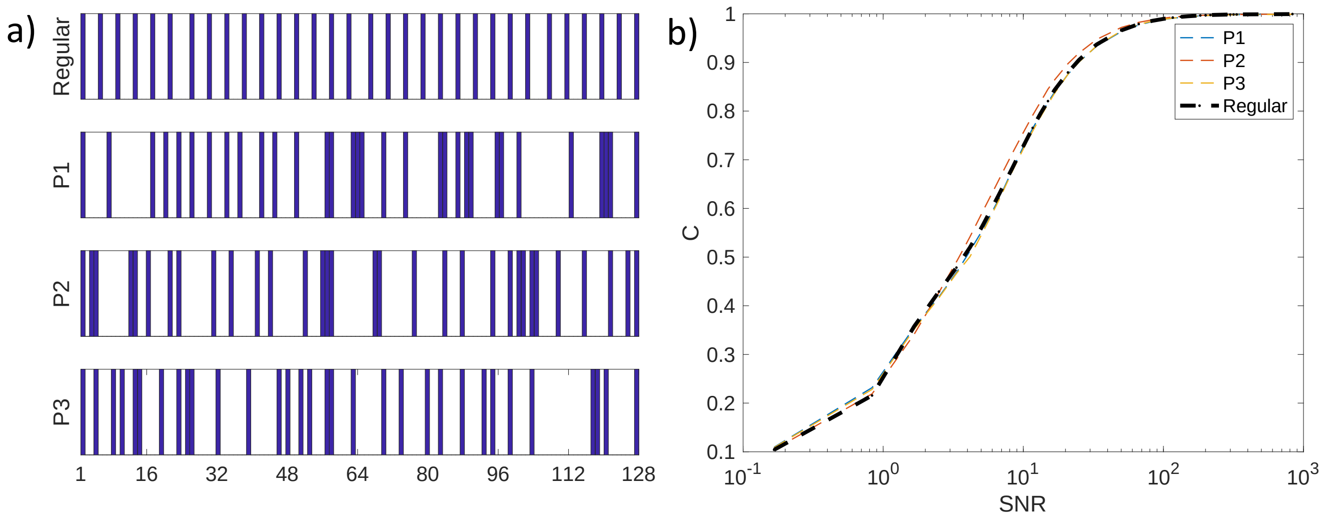

Supplementary Figure 2. Simulation results for different distributions of 32 elements on the transducer's aperture. (a) - A regular and random (P1, P2, P3) distributions of transducer elements. (b) - Average correlation between the reconstructed objects and the modelled ideal object for different values of the SNR. The way of distributing elements does not influence the reconstruction quality.

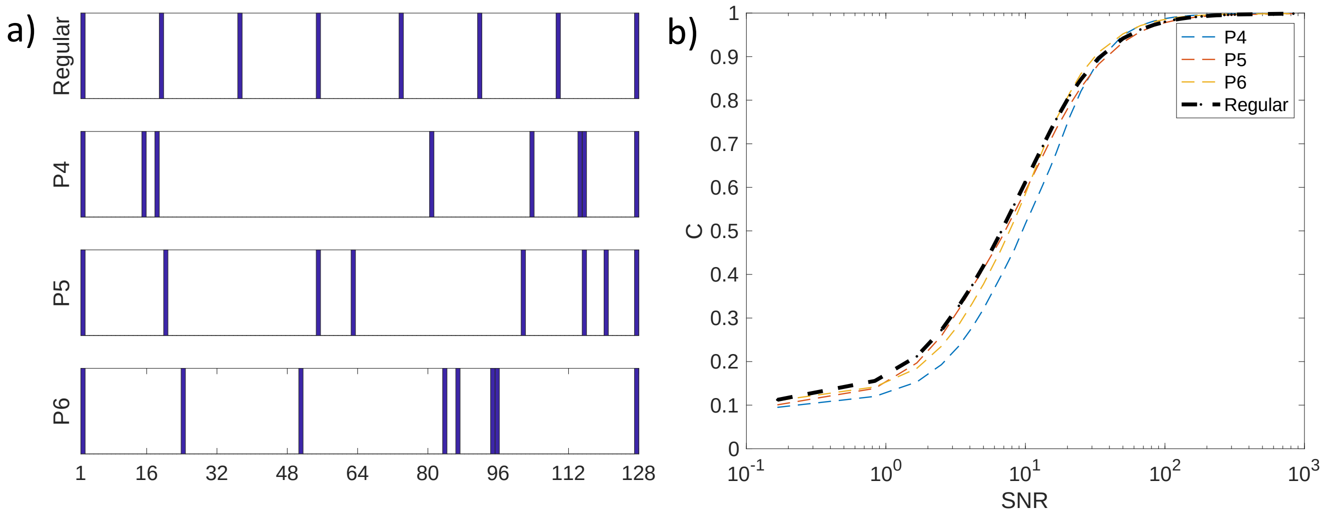

Supplementary Figure 3. Simulation results for different distributions of 8 elements on the transducer's aperture. (a) - A regular and random (P4, P5, P6) distributions of transducer elements. (b) - Average correlation between the reconstructed objects and the modelled ideal object for different values of the SNR. As in Supplementary Figure 2, the way of distributing elements does not influence the reconstruction quality.
